# Synergistic Antifungal Activity of Terbinafine in Combination with Light-Activated Gelatin–Silver Nanoparticles Against *Candida albicans* Strains

**DOI:** 10.3390/pharmaceutics17010125

**Published:** 2025-01-17

**Authors:** Atif Ullah, Fawad Ali, Farman Ullah, Sajid Khan Sadozai, Saeed Ahmed Khan, Sajid Hussain, Abdulwahed Fahad Alrefaei, Sajid Ali

**Affiliations:** 1 Department of Pharmacy, Kohat University of Science and Technology, Kohat 26000, Pakistanfawadali@kust.edu.pk (F.A.); sajidsadozai@kust.edu.pk (S.K.S.); saeedkhanphd@gmail.com (S.A.K.); hussainsajid@kust.edu.pk (S.H.); 2 Department of Zoology, College of Science, King Saud University, P.O. Box 2455, Riyadh 2455, Saudi Arabia; afrefaei@ksu.edu.sa; 3 Department of Horticulture and Life Science, Yeungnam University, Gyeongsan 38541, Republic of Korea

**Keywords:** *Candida albicans*, infection, Terbinafine, silver nanoparticles, synergism, human health

## Abstract

The development of resistance to traditional antifungal therapies has necessitated the exploration of alternative treatment strategies to effectively manage fungal infections, particularly those induced by *Candida albicans* (*C. albicans*). This research investigates the possibility of integrating silver nanoparticles (AgNPs) with Terbinafine to improve antifungal effectiveness. Terbinafine, while potent, faces challenges with specific fungal strains, highlighting the need for strategies to enhance its treatment efficacy. Silver nanoparticles were produced through a light-activated, gelatin-based method, resulting in particle sizes ranging from 56.8 nm to 66.2 nm, confirmed by dynamic light scattering and scanning electron microscopy. Stability studies indicated that AgNPs produced with 30 mg of silver nitrate (AgNO₃) exhibited the greatest stability over 60 days across different temperature conditions. The analysis through UV-visible spectrophotometry revealed a notable shift in the absorption spectra as AgNO₃ concentrations increased, which was associated with a strengthening of plasmon resonance. The effectiveness of the AgNPs and Terbinafine combination was assessed against three strains of *C. albicans* (ATCC 10231, ATCC 90028, and ATCC 18804). Terbinafine demonstrated strong antifungal properties with minimum inhibitory concentrations (MIC) values ranging from 2–4 µg/mL, whereas AgNPs on their own displayed moderate effectiveness. The integrated formulation notably enhanced effectiveness, especially against strain ATCC 90028, revealing a synergistic effect (FIFi = 0.369). These results were complemented by the findings of the time-to-kill assay, where the same strain showed a 3.2 log₁₀ CFU/mL decrease in viable cell count. The process by which AgNPs boost activity entails the disruption of the fungal cell membrane and its internal components, probably as a result of silver ion release and the generation of free radicals. The results indicate that the combination of Terbinafine and AgNPs may act as a powerful alternative for addressing resistant fungal infections, presenting an encouraging direction for future antifungal treatments.

## 1. Introduction

Fungal infections are among the most prevalent global health issues, with over a billion individuals suffering from skin, nail, and hair mycoses, particularly in less-developed nations [1,2]. *C. albicans,* a common element of the human microbiota, can trigger opportunistic diseases, especially in immunocompromised individuals, leading to candidiasis —chronic infections affecting the mouth, skin, or genitourinary tract [3,4].

Current treatments for *C. albicans* infections include topical agents like chlorohexidine and nystatin, as well as systemic medications such as terbinafine, fluconazole, and novel echinocandins [5]. However, the increasing resistance of *Candida* to antifungal drugs necessitates the development of new therapeutic options [6]. Terbinafine, an allylamine antimycotic, is used both orally and topically to treat fungal infections of the skin, nails, and hair. Its mechanism of action involves inhibiting fungal squalene epoxidase, resulting in ergosterol deficiency and squalene accumulation in dermatophytes, molds, and yeasts [7].

Previous research has shown that Terbinafine generally demonstrates limited inhibitory effects against *Candida* species in vitro, except *Candida parapsilosis*, which exhibits relatively low MICs [8]. For *C. albicans*, MIC values for Terbinafine are commonly reported at 4 μg/mL or higher [9,10]. More recent studies confirm the existence of Terbinafine-resistant *C. albicans* clinical isolates, particularly among strains that form biofilms, which further challenges treatment due to decreased antifungal effectiveness [11,12]. Despite the absence of established clinical breakpoints for terbinafine, these observations emphasize the necessity of supplementary or alternative treatments to enhance outcomes against resistant *Candida* infections [13].

The use of AgNPs in combination with existing antimicrobials to enhance their efficacy has garnered significant attention [14]. Recent research indicates that AgNPs combined with antibiotics can reverse antimicrobial resistance [15]. Improved antimicrobial activity against gram-positive and gram-negative bacteria has been observed when antibiotics are used in conjunction with AgNPs [16,17]. While numerous studies have explored the combination of AgNPs with bacteriostatic agents, research on fungistatic remains limited [18]. Furthermore, the eco-friendly production of AgNPs utilizing plant extracts, microorganisms, and other natural sources provides a sustainable and ecologically responsible method [19,20]. In contrast to traditional chemical synthesis techniques, green synthesis reduces the use of harmful chemicals and byproducts, resulting in nanoparticles that exhibit improved stability and compatibility with biological systems [21,22]] Silver nanoparticles (Ag NPs) that respond to external stimuli are becoming increasingly important in the treatment of fungal infections. Their antifungal properties can be enhanced through the use of external triggers like light or ultrasound, which often work by generating reactive oxygen species (ROS) or improving penetration [23]. The effectiveness and specificity of Ag NPs can be further increased by attaching antifungal agents to them, providing a targeted strategy to address fungal resistance [24]. These advancements represent a promising new direction in the field of antifungal therapy [25,26]

The objective of this study is to develop an effective and economical method for producing stable light-activated gelatin-AgNPs and using them in combination with an antifungal drug Terbinafine against various candida species. The results are anticipated to provide fresh perspectives on cutting-edge antifungal treatments and lay the groundwork for further investigation into nanotech-based therapies as powerful alternatives to conventional antifungal medications.

## 2. Material and Methods

### 2.1. Materials

Terbinafine was acquired from Medicraft Pharmaceuticals (Pvt Ltd. Peshawar Pakistan). Various strains of *C. albicans* ATCC 10231, ATCC 90028, and ATCC 18804 were cryopreserved at − 80 °C and were revived before the assay in the laboratory at the Department of Pharmacy, Kohat University of Science and Technology Kohat. Silver nitrate was obtained from Sigma-Aldrich Biochemie GmbH, Steinheim, Germany, gelatin was sourced from Merck, Darmstadt, Germany, Resazurin Dye (R7017) was acquired from Sigma-Aldrich Biochemie GmbH, Steinheim, Germany phosphate buffer saline solution was freshly prepared in the laboratory, and 0.5 McFarland solution and Sabouraud Dextrose Agar (SDA) were procured from Merck. RPMI-1640 medium was purchased from Gibco, Thermo Fisher Scientific, Waltham, MA, USA. All chemicals used were of analytical grade.

### 2.2. Synthesis of Silver Nanoparticles

AgNPs were synthesized using a mixture of AgNO_3_ and 1% gelatin solution. The AgNO_3_ solution was prepared by combining 10–30 mg of AgNO_3_ with 1 mL of distilled water and stirring magnetically. To prepare a 1% gelatin solution, 1.0 gm of gelatin was dissolved in 100 mL of water using a magnetic stirrer and gentle heating to 60 °C for complete dissolution. The synthesis process involved adding 1 mL of the AgNO_3_ solution to 10 mL of the gelatin solution using a micropipette while stirring magnetically at 600–800 rpm. The mixture was then left on the magnetic stirrer for 24 h under light exposure [27,28]. The resulting golden-colored AgNPs were subsequently analyzed using Dynamic Light Scattering (DLS) techniques.

### 2.3. Characterization of Silver Nanoparticles

#### 2.3.1. Dynamic Light Scattering (DLS) Studies

DLS studies were performed to measure the mean particle size and polydispersity index (PDI) with a Zeta sizer Nano ZS (Malvern Instruments, Malvern, UK). From each formulation, 50 µL of a dispersion of freshly prepared nanoparticles was diluted with 1.0 mL distilled water at 25 °C with a back scattering angle of 172° [29].

#### 2.3.2. Scanning Electron Microscopy (SEM)

The morphology, particle size, and size distribution of nanoparticles were examined using SEM (EVO HD 15, Carl Zeiss Microscopy GmbH., Jena, Germany). To ensure adequate conductivity, samples were covered with a thin gold layer (Q150RES, Quorum Technologies Ltd., East Grinstead, UK). Images were captured at an acceleration voltage of 5 kV [30].

#### 2.3.3. UV-Visible Spectroscopy

UV-Visible absorption spectroscopy was employed for optical characterization of the colloidal suspensions. The analysis was conducted using a UV/Vis spectrophotometer (Jasco V-530 Jasco Corporation, Tokyo, Japan). Ethanol served as the reference for obtaining UV-Vis spectra. The colloidal stability of AgNPs over time was evaluated by visual observation and UV-Vis spectroscopy [31].

#### 2.3.4. Stability Studies of Silver Nanoparticles

Stability studies were conducted at various temperatures and time intervals. The particle size and PDI were analyzed using a Malvern zeta sizer (Malvern Panalytical, Malvern, UK). Each formulation was subjected to different temperature conditions (2–4 °C, 28 °C, and 40 °C) to evaluate the effects of temperature and time on the stability of the prepared nanoparticles. Samples from each formulation at different temperature conditions were withdrawn and analyzed on days 1, 15, 30, 45, and 60 [32,33,34]. Following the stability studies, the most stable formulation was selected as the optimized formulation for further investigations.

### 2.4. Minimum Inhibitory Concentration (MIC)

The MIC was determined to assess the inhibitory effects of Terbinafine and synthesized AgNPs. This evaluation employed the broth microdilution technique, following the guidelines outlined in the CLSI M27-A3 document (Clinical and Laboratory Standards Institute 2008), with adjustments as suggested by Rojas et al. (2014).

The preparation of all inoculum suspensions involved using sterile saline solutions, with the turbidity adjusted to match a 0.5 McFarland scale using a densitometer. Subsequently, this inoculum underwent a 1:100 dilution in RPMI-1640 medium supplemented with 2% glucose buffered with MOPS (3-(N-morpholino) propanesulphonicacid) for the growth of *C. albicans*, resulting in a final concentration ranging from 0.5 to 2.5 × 10^5^ CFU/mL [35].

### 2.5. In Vitro Antifungal Activity

To evaluate the antifungal efficacy of Terbinafine and AgNPs against various strains of *C. albicans* (ATCC 10231, ATCC 18804, and ATCC 90028), RPMI-1640 medium was used with the supplemented components as discussed above in 96-well microplates. The medium was mixed with *C. albicans* culture and the Terbinafine and AgNPs solution, followed by a 2-day incubation period. The MIC value was determined by identifying the lowest concentration of Terbinafine and AgNPs at which fungal growth was inhibited. Terbinafine concentrations varied from 32 to 0.06 µg/mL, whereas AgNPs were evaluated at doses ranging from 250 to 0.45 µg/mL. To guarantee the experiment’s validity, both growth and sterility controls were included. Fluconazole (128 μg/mL) was used as the reference standard [36].

### 2.6. Synergism

#### 2.6.1. Checkerboard Survival Assay

A checkerboard survival assay was employed to evaluate the MIC and potential synergistic effects of Terbinafine and AgNPs. The process involved preparing 2-fold dilutions of each particle dispersion in PBS and applying them along the *x*-axis (Terbinafine) and *y*-axis (AgNPs) of a 96 well plate. The desired checkerboard pattern was achieved by combining each dilution of both substances [37].

Following the addition of 10^5^ *C. albicans* cells per well, the plates were incubated for 48 h at 30 °C. The final drug concentrations tested ranged from 32 µg/mL to 0.06 µg/mL for Terbinafine and 250 µg/mL to 0.45 μg/mL for AgNPs. Cell viability was assessed by transferring 5 μL from each well onto SDA plates. After incubation for 2 days at 30 °C, potential cell growth was documented. DMSO dilutions served as controls.

To assess the effects of the combinations, the Fractional Inhibitory Concentration Index (FIC*i*) was calculated using a specific equation.

FIC*i* = FIC_Terbinafine_ + FIC_AgNPs_

FIC _Terbinafine_ = MIC of Terbinafine in combination/MIC of Terbinafine.

FIC_AgNPs_ = MIC of AgNPs in combination/MIC of AgNPs alone.

An FIC index value in the range of ≤0.5 indicates synergism between two tested compounds, an additive or indifferent effect is denoted by an FIC*i* value between 0.5 and 4.0, and antagonism is indicated when the value of FIC*i* is above 4.0 [38].

#### 2.6.2. Time to Kill Assay Method

Time–kill assay experiments were conducted by inoculating the *C. albicans* strains ATCC 10231, ATCC 18804, and ATCC 90028 into potato dextrose agar (PDA) medium at a final concentration of 4.5 × 10⁴ cells/mL. PDA medium was chosen because it supports fungal growth over extended periods, which is essential for monitoring time–kill dynamics. A 5 mL aliquot of each culture was dispensed into tubes containing Terbinafine, DMSO (as a negative control), AgNPs, and a combination of Terbinafine and AgNPs. After adding varying concentrations of the drugs or combinations, the tubes were incubated at 30 °C with shaking at 220 rpm. At set intervals (0, 12, 24, 48, and 72 h), 100 µL samples were diluted in 1× PBS and streaked onto SDA plates. Yeast colonies were counted after 2–3 days of incubation at 30 °C, and the results were plotted as the mean colony count (log_10_ CFU/mL) [39].

The fungicidal activity was defined as a reduction of ≥3 log_10_ CFU/mL from the initial inoculum. In contrast, fungistatic activity was identified by maintaining or reducing the count by less than 3 log_10_ CFU/mL. Synergistic and antagonistic effects were evaluated by plotting the relative viable counts over time on a logarithmic scale. Antagonism was indicated by a ≥ 2 log_10_ increase in CFU/mL compared to the least active agent, while synergism was identified by a ≥ 2 log_10_ decrease compared to the most active agent. An additive effect was defined as a decrease of 1 to 2 log_10_ CFU/mL relative to the most active agent [40].

### 2.7. Statistical Analysis

Statistical analysis was performed to ensure accuracy and reliability, with the results expressed as mean ± standard deviation (SD). The criterion for statistical significance was set at *p* < 0.5, and data analysis was conducted using Graph Pad Prism software (Version 5).

## 3. Results and Discussion

To overcome the challenges faced in treating fungal infections, conventional antifungal therapies have become increasingly limited due to rising drug resistance and frequent treatment failures against various fungal strains [41]. These issues have prompted the exploration of alternative therapies to provide more effective and optimal patient care, aiming to reduce the prolonged treatment periods commonly associated with fungal infections. One promising approach is the combination of AgNPs with the antifungal drug terbinafine. Terbinafine, though effective, is not typically the first choice for infections caused by certain strains, such as *C. albicans*, due to its limited efficacy in some cases [42]. The objective of this study was to address these limitations by formulating a combination of Terbinafine with AgNPs, which was synthesized using a simple gelatin-based procedure and activated by light. This method leverages the enhanced antifungal potential of silver nanoparticles, offering a synergistic effect that may improve Terbinafine’s effectiveness and broaden its therapeutic applicability across resistant fungal strains.

### 3.1. Synthesis of Silver Nanoparticles

AgNPs were synthesized using AgNO₃ and a 1% gelatin solution as the stabilizing agent. The formation of golden-colored AgNPs indicates successful nanoparticle synthesis [43].

### 3.2. Dynamic Light Scattering Studies

**Figure 1:**
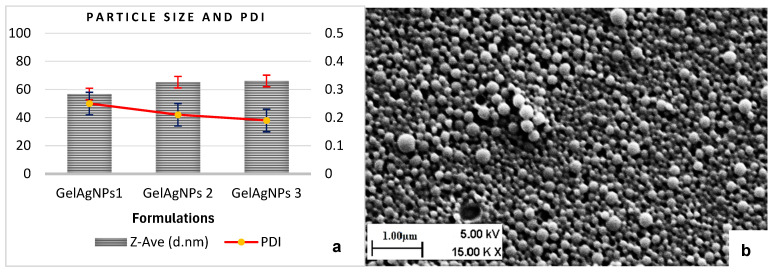
(**a**) DLS studies showing particle size and PDI value at 28 °C. (**b**) SEM image of 1% gelatin AgNPs.

DLS studies were performed, and all the formulations exhibited an acceptable particle size range of 56.8 ± 2.7 nm to 66.2 ± 1.1 nm at 28 °C. Additionally, the PDI values were in the range of 0.21 to 0.25, as shown in Figure 1a. These values indicate that the formulations maintain uniform size and distribution, which are crucial for their stability and effectiveness. Initially, the formulation with the least amount of AgNO_3_ showed the smallest particle size, and the formulation with a higher AgNO_3_ amount showed a slightly larger particle size. However, this trend shifted over time, and it was noted that the AgNP formulation using 30 mg AgNO_3_ demonstrated the highest stability. This formulation maintained its particle size within the specified range under various temperature conditions for a period of 60 days. This phenomenon is elaborated upon in the stability studies section focusing on silver nanoparticles.

### 3.3. Scanning Electron Microscopy

The SEM images showed particles with uniform, spherical shapes. The nanoparticles (NPs) exhibited sizes ranging from 50 nm to 65 nm, with distinct and uniform boundaries and no visible aggregation (Figure 1b). These observations aligned with the measurements obtained through dynamic light scattering, as presented in Figure 1.

### 3.4. UV-Visible Spectrophotometry

**Figure 2:**
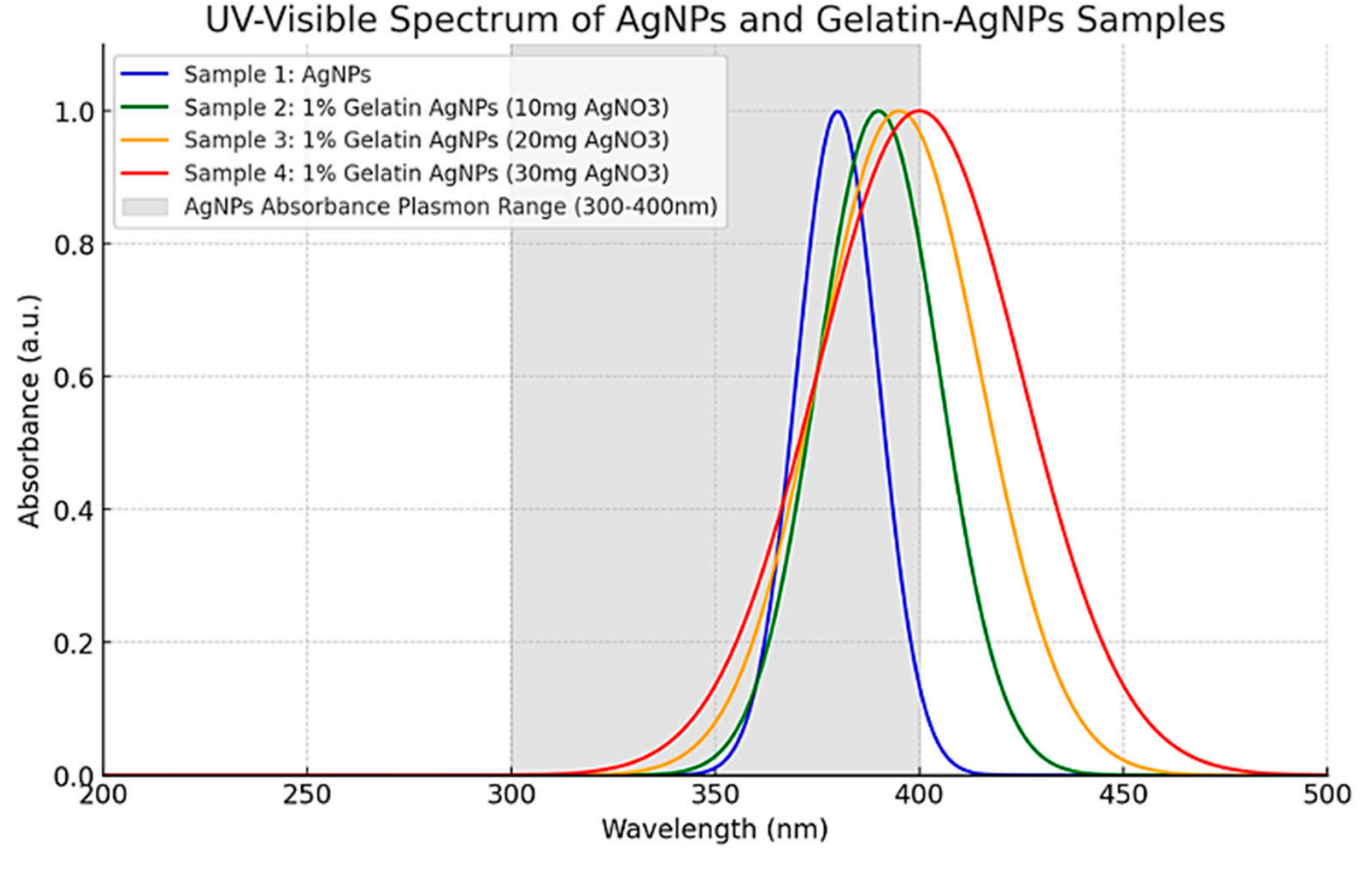
UV-Visible absorption spectra of AgNPs and gelatin-stabilized AgNPs at different AgNO₃ levels. Sample 1 (blue): commercial AgNPs, Sample 2 (green): gelatin-stabilized AgNPs (1% gelatin, 10 mg AgNO₃),Sample 3 (orange): gelatin-stabilized AgNPs (1% gelatin, 20 mg AgNO₃), Sample 4 (red): gelatin-stabilized AgNPs (1% gelatin, 30 mg AgNO₃). The shaded region reveals AgNPs’ 300–400 nm plasmon resonance. Due to particle size or aggregation, higher silver nitrate concentrations redshift the plasmon peak.

A UV-Visible spectrophotometer was used to analyze and compare the spectrum of commercially available AgNPs with AgNPs prepared from 1% gelatin solution in the presence of light. Four samples were analyzed in triplicate. The synthesis of 1% gelatin AgNPs was confirmed from the UV-Visible spectrum as shown in Figure 2.

The spectral analysis reveals distinct characteristics for each sample. Sample 1 (AgNPs) exhibits a relatively narrow peak at approximately 380 nm. In contrast, Sample 2 (1% gelatin AgNPs, 10 mg AgNO₃) displays a slightly wider peak, shifted to about 390 nm. Sample 3 (1% gelatin AgNPs, 20 mg AgNO₃) demonstrates a further shift to roughly 395 nm, accompanied by increased peak broadening. Sample 4 (1% gelatin AgNPs, 30 mg AgNO₃) shows the widest peak, centered around 400 nm.

This comparative analysis indicates that the introduction of gelatin and the escalation of AgNO₃ concentration led to two observable effects: a red shift (movement towards higher wavelengths) and peak broadening. These phenomena can be attributed to alterations in the surrounding gelatin matrix, which influence the surface plasmon resonance of the AgNPs. The typical plasmon resonance range for AgNPs, spanning from 300 to 400 nm, is denoted by the gray shaded area in the spectrum [44].The AgNPs were characterized using DLS to determine their size distribution. The results confirmed the formation of nanoparticles within the expected size range, with uniform distribution and stability provided by the gelatin matrix. The successful synthesis was further evident due to the change in color of the solution, commonly associated with AgNP formation due to surface plasmon resonance.

In the synthesis of AgNPs, light provides energy to reduce silver ions (Ag⁺) from AgNO₃ to metallic silver (Ag⁰). This light-driven process excites electrons, allowing silver ions to gain electrons from a reducing agent (such as gelatin), forming silver atoms that cluster into nanoparticles. Light ensures uniform reduction and promotes the growth and stability of these nanoparticles. Surface Plasmon Resonance (SPR), caused by light interaction with the particles, gives them their golden color. Light acts as a catalyst, driving the reaction and controlling nanoparticle size and stability [45,46].

### 3.5. Stability Studies of Gelatin AgNPs

**Figure 3:**
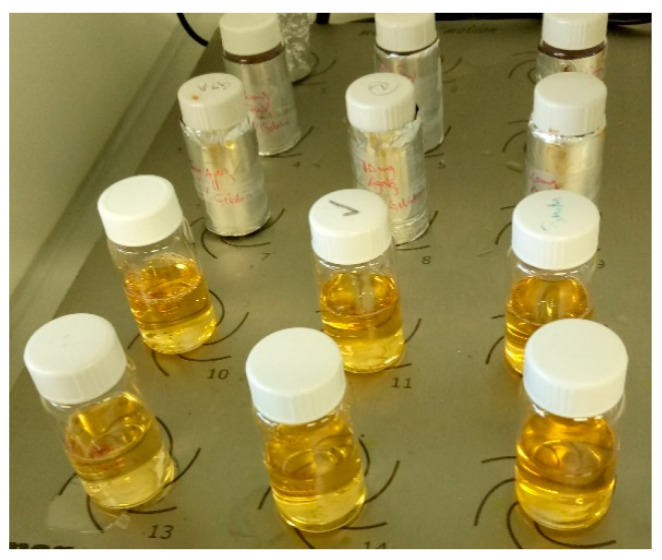
Stability studies of 1% gelatin AgNPs of various formulations.

The stability studies were carried out at three different temperature conditions, i.e., 2 °C–8 °C, 28 °C, and 40 °C, for 60 days, and samples were analyzed for size (nm) and PDI values. The DLS results showed that the 10 and 20 mg AgNO_3_ formulations were less stable as compared to 30 mg AgNO_3_ due to an imbalance between silver ions and stabilizing gelatin. The lower silver concentration in the 10 mg solution led to insufficient nucleation, irregular particle growth, and inadequate capping by gelatin, causing instability. In contrast, the formulation containing 30 mg of AgNO_3_ showed stable particle sizes over 60 days at 2–8 °C, with sizes ranging from 65.6 ± 1.7 nm to 78.3 ± 1.5 nm and PDI values increasing from 0.17 to 0.25. The *p-value* of 0.402 (*p* > 0.5) indicated no significant changes, confirming stability at this temperature. At 25–28 °C, the particle size ranged from 66.2 ± 1.1 nm to 79.1 ± 1.8 nm, with PDI values between 0.19 to 0.28. The *p-value* of 0.262 (*p* > 0.5) indicated no significant changes, suggesting stability at this moderate temperature. At 40 °C, the particle size increased from 66.8 ± 1.9 nm to 81.2 ± 1.9 nm, and PDI values increased from 0.20 to 0.29. Despite the noticeable change, the *p*-value of 0.48 (*p* > 0.5) confirmed that the changes were not statistically significant, indicating stability even at higher temperatures [47], which is evident from the data shown in the Table 1 and Figure 3.

### 3.6. In Vitro Antifungal Activity

The investigation evaluated the efficacy of Terbinafine and AgNPs against three strains of *C. albicans* as shown in Table 2.

The antifungal action of AgNPs is attributed to the release of silver ions in aqueous settings. These ions engage with the negatively charged external surfaces of *Candida albicans* cells, resulting in modifications to the physical and chemical properties of the cell membrane. This interaction interferes with critical cellular functions, such as permeability, respiration, and osmoregulation. Moreover, AgNPs may damage DNA, proteins, and other cellular constituents, thereby diminishing the organism’s reproductive ability. These processes together improve the antifungal effects, especially when AgNPs are used with Terbinafine [48].

The antifungal efficacy of Terbinafine and AgNPs was assessed against *C. albicans* strains ATCC 10231, ATCC 90028, and ATCC 18804. Terbinafine exhibited potent inhibition, with MIC values ranging from 2 to 4 µg/mL, while AgNPs showed moderate efficacy, with MIC values between 62.5 and 250 µg/mL. The combination of Terbinafine with AgNPs improved effectiveness, especially against ATCC 18804. These findings underscore the efficacy of combining Terbinafine with AgNPs to enhance therapy for *C. albicans* infections (Table 3).

These results corroborate prior research, revealing that AgNPs limit the development of *C. albicans*, with differential efficiency across strains. Consistent with observations in other fungi such as *Sclerotinia sclerotiorum* and *Alternaria alternata*, AgNPs compromise fungal cell membranes, likely inducing cell death by poration. Antibacterial action may be associated with the production of free radicals that harm DNA and proteins, as shown by previous research. These mechanisms augment the antifungal efficacy reported in our Terbinafine–AgNP combination [49].

### 3.7. Checkerboard Survival Assay

The combined effects of Terbinafine and AgNPs were tested against *C. albicans* ATCC 10231, ATCC 90028, and ATCC 18804. Terbinafine and AgNPs were combined in different concentrations in each well, and yeast cells were added. Growth inhibition and lethal effects induced by the drug compounds were identified by observing colony formation of yeast cells, and the respective MIC values were calculated as shown in Table 4.

As demonstrated in Table 4, the FICi values for Terbinafine and AgNPs were less than 0.5, indicating a potent synergistic effect when these were used in combination against various strains of *C. albicans*. The *C. albican* ATTC90028 showed strong synergistic characteristics (FICi 0.369) as compared to other two strains (FICi 0.379 and 0.388, respectively). These findings not only emphasize the unique antifungal properties of the examined Terbinafine, but also align with previously documented synergistic effects between AgNPs and other azole-based antifungal medications, including fluconazole and voriconazole.

The demonstrated synergistic effect between Terbinafine and silver nanoparticles (AgNPs) in combating *Candida albicans* strains illustrates the promising potential of integrating nanotechnology with antifungal medications to improve treatment outcomes. The notably low FICi values (below 0.5) indicate a substantial decrease in the required concentrations of both agents, potentially reducing toxic effects. AgNPs likely boost Terbinafine’s effectiveness by compromising fungal cell membranes and producing reactive oxygen species, thereby increasing the fungi’s vulnerability to ergosterol synthesis inhibition. These results are consistent with earlier research on the synergistic impact of AgNPs combined with azole antifungals, suggesting that this combination could offer an effective strategy for addressing antifungal resistance and enhancing treatment efficacy, particularly in resistant strains.

### 3.8. Time to Kill Assay Method

To validate the synergistic effect of Terbinafine and AgNPs in combination, as observed in the checkerboard survival assay, a time to kill assay method was employed. *C. albicans* strains were prepared at a final concentration of 4.5 × 10^4^ cells/mL in potato dextrose medium. Five-milliliter aliquots were distributed into culture tubes containing various treatments: DMSO (positive control), Terbinafine (1 × MIC), AgNPs (1⁄2 × MIC), and a combination of Terbinafine (1 × MIC) with AgNPs (1⁄2 × MIC). Following the addition of samples at their respective concentrations, the tubes were incubated at 30 °C and 220 rpm. At specified intervals (0, 12, 24, 48, 72 h), 100 µL samples were extracted from each tube, diluted in 1 × PBS, and plated on SDA. After incubating at 30 °C for 2–3 days, yeast colonies were enumerated.

**Figure 4:**
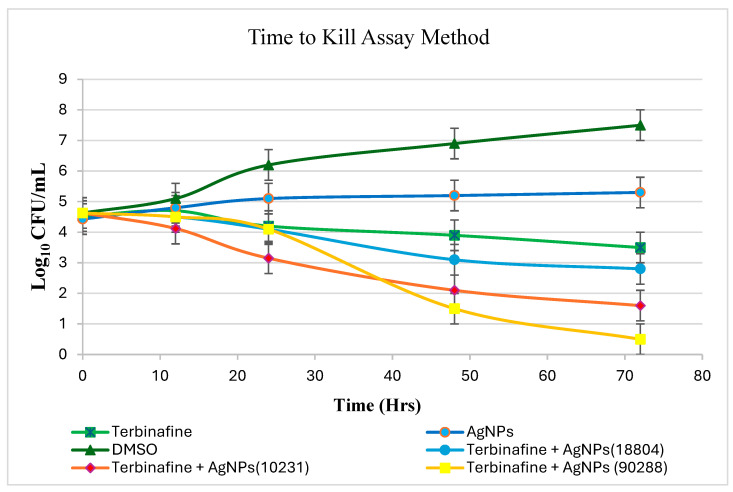
The time to kill assay graph compares Terbinafine (green squares), DMSO (green triangles), AgNPs (blue circles), and Terbinafine with AgNPs for *Candida albicans* ATCC 18804 (yellow squares with lines). Microbial viability decreased considerably over 70 h with the combined treatment, showing synergy.

The time–kill assay method was employed to assess the combined impact of Terbinafine and AgNPs on cell viability, supporting the results obtained from the checkerboard survival assay. The combination of Terbinafine and AgNPs Against *C. albicans* 90288 exhibited fungicidal activity, demonstrated by a reduction of ≥3 log_10_ CFU/mL from the initial inoculum. Similar fungicidal effects were noted for *C. albicans* strains 10231 and 18804, albeit with smaller decreases in CFU/mL compared to strain 90288, as shown in Figure 4. In contrast, Terbinafine alone produced less than a 1 log_10_ reduction in CFU/mL, while AgNPs and DMSO individually showed increased log_10_ CFU/mL values, indicating no antifungal activity when used independently.

Starting with an initial inoculum of 4.5 × 10^4^ cell/mL, Terbinafine (1 × MIC) and AgNPs (1⁄2 × MIC), when administered separately, displayed minimal antifungal effects against *C. albicans* strains at 72 h. However, their combined use resulted in substantial decreases in log_10_ CFU/mL compared to Terbinafine alone: 3.2 for strain 90288, 2.2 for strain 10231, and 1.5 for strain 18804. When used individually, AgNPs and DMSO led to 2.4 and 4.7 increases in log_10_ CFU/mL, respectively, in comparison to the Terbinafine and AgNPs combination, which demonstrated the least fungicidal activity against *C. albicans* strain 18804, as illustrated in Figure 4.

The time–kill assay results further validate the potent synergistic effect of Terbinafine and AgNPs, as demonstrated by the significant reduction in CFU/mL compared to their individual treatments. The combination therapy achieved fungicidal activity with reductions of ≥3 log10 CFU/mL for *C. albicans* strains, particularly ATCC 90288, highlighting its enhanced efficacy. This pronounced activity suggests that AgNPs enhance the action of Terbinafine by disrupting fungal cell integrity, facilitating increased drug penetration, and generating reactive oxygen species, which together accelerate fungal cell death. These findings reinforce the therapeutic potential of combining antifungal drugs with nanomaterials, particularly in addressing the limitations of single-agent therapies. The results also highlight the potential of this combination to minimize drug resistance and reduce the required drug doses, offering a safer and more effective approach for managing *C. albicans* infections.

## 4. Conclusions

This study demonstrates the promising synergistic effects of Terbinafine when combined with light-activated gelatin–silver nanoparticles against Candida albicans strains. The results from the checkerboard survival assay and time–kill assay method clearly show that this combination not only enhances the antifungal activity of Terbinafine, but also effectively inhibits fungal growth at lower concentrations compared to the agents used independently. These findings suggest that the combination of AgNPs with Terbinafine offers a potential strategy to improve treatment outcomes for superficial Candida infections while reducing the required dosage of Terbinafine.

The experimental data highlight the significant potential of AgNPs in improving the efficacy of allylamine drugs like Terbinafine. Furthermore, the synergistic interaction between AgNPs and antifungal agents presents a novel approach to combat fungal infections, especially in the context of increasing resistance and therapy failure. Our results underscore the importance of developing such nanoparticle-based formulations to address the challenges of drug resistance and enhance the effectiveness of existing antifungal therapies.

## Figures and Tables

**Table 1:** Measurements of the sizes of AgNPs for 60 days at various temperatures.

S.NO	1% Gelatin	AgNO_3_ (mg)	Average Particle Sizes of AgNPs ± SD											
			Day 0		Day 1		Day 15		Day 30		Day 45		Day 60	
Particle size (nm) stability studies at 2–8 °C														
			Z- Ave (nm)	P.D.I	Z-Ave (nm)	P.D.I	Z-Ave (nm)	P.D.I	Z-Ave (nm)	P.D.I	Z-Ave (nm)	P.D.I	Z-Ave (nm)	P.D.I
1	1 g	10 mg	55.9 ± 2.8	0.21	60.6 ± 2.7	0.25	64.4 ± 2.9	0.29	90.1 ± 2.2	0.38	110.2 ± 2.3	0.41	130.3 ± 2.9	0.48
2	1 g	20 mg	62.2 ± 2.4	0.18	63.9 ± 2.9	0.24	69.1 ± 2.5	0.28	80.8 ± 2.1	0.35	90.5 ± 2.0	0.40	110.8 ± 3.5	0.41
3	1 g	30 mg	65.6 ± 1.7	0.17	66.5 ± 1.5	0.21	69.8 ± 1.4	0.22	72.1 ± 2.0	0.21	74.4 ± 1.6	0.23	78.3 ± 1.5	0.25
Particle size (nm) stability studies at 25–28 °C														
1	1 g	10 mg	56.8 ± 2.7	0.25	62.5 ± 3.5	0.25	65.2 ± 3.1	0.29	95.1 ± 2.3	0.31	116.4 ± 2.5	0.44	140.1 ± 3.6	0.51
2	1 g	20 mg	65.2 ± 3.1	0.20	68.9 ± 3.1	0.24	73.1 ± 3.0	0.28	90.8 ± 2.1	0.30	93.5 ± 2.7	0.41	120.8 ± 2.9	0.45
3	1 g	30 mg	66.2 ± 1.1	0.19	66.9 ± 2.0	0.21	70.8 ± 2.3	0.22	73.1 ± 1.9	0.24	76.2 ± 2.0	0.25	79.1 ± 1.8	0.28
Particle size (nm) stability studies at 40 °C														
1	1 g	10 mg	59.9 ± 3.1	0.28	68.6 ± 4.0	0.29	75.4 ± 3.8	0.32	98.1 ± 2.5	0.35	116.2 ± 3.1	0.48	150.3 ± 4.5	0.68
2	1 g	20 mg	65.2 ± 2.5	0.25	69.9 ± 3.8	0.27	73.1 ± 3.0	0.29	93.8 ± 2.3	0.31	110.5 ± 2.9	0.41	130.8 ± 3.2	0.54
3	1 g	30 mg	66.8 ± 1.9	0.20	68.4 ± 2.1	0.21	71.5 ± 1.9	0.22	75.1 ± 2.0	0.25	79.1 ± 2.2	0.26	81.2 ± 1.9	0.29

Z-Ave (nm) refers to the average particle size of AgNPs (AgNPs). PDI stands for polydispersity index, indicating the distribution of particle sizes. Data show particle size increases and stability changes over time at various temperatures.

**Table 2:** Terbinafine and silver nanoparticles (µg/mL) were examined in a microtiter plate assay against *Candida strains* (ATCC 10231, 90028, and 18804) using serial dilutions. Rows 11 and 12 define growth and sterility.

Well Number	Terb Against ATCC 10231 (μg/mL)	AgNPs Against ATCC 10231 (μg/mL)	Terb Against ATCC 90028 (μg/mL)	AgNPs Against ATCC 90028 (μg/mL)	Terb Against ATCC 18804 (UG/ML)	AGNPS Against ATCC 18804 (UG/ML)
1	32	250	32	250	32	250
2	16	125	16	125	16	125
3	8.0	62.5	8.0	62.5	8.0	62.5
4	4.0	31.2	4.0	31.2	4.0	31.2
5	2.0	15.6	2.0	15.6	2.0	15.6
6	1.0	7.8	1.0	7.8	1.0	7.8
7	0.5	3.9	0.5	3.9	0.5	3.9
8	0.25	1.9	0.25	1.9	0.25	1.9
9	0.12	0.9	0.12	0.9	0.12	0.9
10	0.06	0.45	0.06	0.45	0.06	0.45
11	Growth controls	Growth controls	Growth controls	Growth controls	Growth controls	Growth controls
12	Sterility	Sterility	Sterility	Sterility	Sterility	Sterility

* Terbinafine (Terb).

**Table 3:** A microtiter plate assay was used to investigate Terbinafine (Terb) and silver nanoparticles (AgNPs) (µg/mL) against *Candida albicans* strains (ATCC 10231, ATCC 18804, and ATCC 90028). The table shows agent concentrations in wells 1–10 for each strain.

Microtiter Plate Well No.		1	2	3	4	5	6	7	8	9	10
*C. albicans* ATCC 10231	**Conc. (µg/mL)**										
	Terbinafine	32	16	8.0	4.0	2.0	1.0	0.50	0.25	0.12	0.06
	AgNPs	250	125	62.5	31.2	15.6	7.8	3.9	1.9	0.9	0.45
*C. albicans* ATCC 18804	Terbinafine	32	16	8.0	4.0	2.0	1.0	0.5	0.25	0.12	0.06
	AgNPs	250	125	62.5	31.2	15.6	7.8	3.9	1.9	0.9	0.45
*C. albicans* ATCC 90028	Terbinafine	32	16	8.0	4.0	2.0	1.0	0.50	0.25	0.12	0.06
	AgNPs	250	125	62.5	31.2	15.6	7.8	3.9	1.9	0.9	0.45

**Table 4:** MICs of Terbinafine and AgNPs against *Candida albicans* strains (ATCC 10231, ATCC 18804, and ATCC 90028) evaluated alone and in combination. All strains showed synergistic interactions between Terbinafine and AgNPs based on their fractional inhibitory concentration (FIC) and FICi values.

Candida Strains.	Sample	MIC (µ/mL)		FIC	FICi	Outcome
		Alone	Combination			
*C. albicans* ATTC10231	Terbinafine	1.0	0.21	0.21	0.379	Synergism
	AgNPs	62.5	10.65	0.169		
*C. albicans* ATTC18804	Terbinafine	0.5	0.12	0.240	0.388	Synergism
	AgNPs	62.5	9.25	0.148		
*C. albicans* ATTC90028	Terbinafine	2.0	0.25	0.125	0.369	Synergism
	AgNPs	62.5	15.25	0.244		

FIC = fractional inhibitory concentration; MICc = MIC of sample in combination, MICo = MIC of sample alone: FIC = MICc/MICo; FICi (FIC index) = FIC of Terbinafine + FIC of AgNPs.

## Data Availability

The authors confirm that the data supporting the findings of this study are available within the article.
